# Supplementary material for: Complexome profiling of the Chlamydomonas psb28 mutant reveals TEF5 as an early PSII assembly factor
Source: Plant Cell. 2025 Mar 17;37(6):koaf055. doi: 10.1093/plcell/koaf055 (PMC12164590; doi:10.1093/plcell/koaf055)
Supplement: koaf055_Supplementary_Data [file koaf055_supplementary_data.zip › psb28_interactive.zip!/plots/intensity/qualityPlots/peptides/_Cre11.g468350.t1.2.html]

### Cre11.g468350.t1.2

Trivial name:   
SeqCoverage [median]: 21.00  
Mass: 64.32 kDa  
  
 

| Sequence | MissedCleavage | Mass [Da] | wtIntensity | muIntensity | Score |
| --- | --- | --- | --- | --- | --- |
| AGDAASVPLVLSSR | 0 | 1341.73 | 0.00 | 5585.48 | 120.77 |
| FMNAGQVLSK | 0 | 1093.56 | 0.00 | 1107.23 | 81.97 |
| GAIAFEELGLTAQR | 0 | 1474.78 | 603.75 | 0.00 | 106.35 |
| IGYSEVIR | 0 | 935.51 | 0.00 | 931.91 | 67.03 |
| QLRPVDSSSLYR | 1 | 1419.75 | 0.00 | 2160.01 | 77.60 |
| SGLAADLVEAAER | 0 | 1300.66 | 2805.02 | 4593.23 | 103.34 |
| SGLAADLVEAAERK | 1 | 1428.76 | 716.99 | 0.00 | 44.19 |
| TLALVLR | 0 | 784.52 | 0.00 | 3198.22 | 94.16 |
| VSFHFESELVR | 0 | 1348.68 | 0.00 | 3406.99 | 66.26 |
|  |  |  | 4125.8 | 20983.1 |  |
